# Association of Two Polymorphisms in *H2B.W* Gene with Azoospermia and Severe Oligozoospermia in An Iranian Population 

**DOI:** 10.22074/ijfs.2015.4241

**Published:** 2015-07-27

**Authors:** Haleh Haji Ebrahim Zargar, Anahita Mohseni Meybodi, Marjan Sabbaghian, Maryam Shahhoseini, Ummulbanin Asadpor, Mohammad Ali Sadighi Gilani, Mohammad Chehrazi, Mansoureh Farhangniya, Seyed Abolhassan Shahzadeh Fazeli

**Affiliations:** 1 Department of Molecular and Cellular Biology, Faculty of Basic Sciences and Advanced Technologies in Biology, University of Science and Culture, Tehran, Iran; 2 Department of Genetics at Reproductive Biomedicine Research Center, Royan Institute for Reproductive Biomedicine, ACECR, Tehran, Iran; 3 Department of Andrology at Reproductive Biomedicine Research Center, Royan Institute for Reproductive Biomedicine, ACECR, Tehran, Iran; 4 Department of Epidemiology and Reproductive Health at Reproductive Epidemiology Research Center, Royan Institute for Reproductive Biomedicine, ACECR, Tehran, Iran; 5 Iranian Biological Resource Center (IBRC), ACECR, Tehran, Iran

**Keywords:** Histone, Male Infertility, Polymorphism

## Abstract

**Background:**

During spermatogenesis, the H2B family, member W (*H2B.W*) gene, en- codes a testis specific histone that is co-localized with telomeric sequences and has the potential role to mediate the sperm-specific chromatin remodeling. Previously *H2B.W* genetic variants were reported to be involved in susceptibility to spermatogenesis im- pairment. In the present study, two single nucleotide polymorphisms (SNPs) in 5΄UTR and exon 1 of *H2B.W* gene were examined to investigate possible association of these polymorphisms with male infertility in Iranian population.

**Materials and Methods:**

This case control study was conducted in Royan institute during four-year period (2010–2013). Genetic alteration of two SNPs loci, −9C>T and 368A>G, in *H2B.W* gene were indicated in 92 infertile men who were divided into two main groups includ- ing azoospermia (n=46) and sever oligozoospermia (n=46), while there was 60 fertile men as control group. Azoosperima was also divided into three sub-groups including sertoli cell only syndrome (SCOS, n=21), complete maturation arrest (CMA, n=17) and hypo spermatogenesis (n=8) according to testicular biopsy. For analysis, polymerase chain reaction-restriction frag- ment length polymorphism (PCR-RFLP) technique was applied.

**Results:**

The frequency of allele −9T was significantly higher in CMA group than in patients with SCOS (P<0.05). The haplotype TA (corresponding to simultaneous occur- rence of −9T and 368A) compared with haplotype CA (corresponding to simultaneous occurrence of −9C and 368A) in patients suffering from CMA significantly increased, compared with patients had SCOS (P<0.05). However, statistical studies indicated that in general, the distribution frequencies of −9C>T and 368A>G had no significant difference between the infertile groups and control (P=0.859 and P=0.812, respectively).

**Conclusion:**

This investigation showed that SNP −9C>T might be contribute to CMA in azoo- spermic patients and SNP 368A>G had no correlation with male infertility in Iranian population.

## Introduction

One of the most common causes of male infertility is impaired spermatogenesis. It is an intricate, temporal process whereby adult stem cells either self-renew or generate daughter cells that are transformed into a specialized testicular spermatozoon (1-3).

Dramatic chromatin remodeling and chromosomes rearrangement can occur during spermatogenesis. These structural alterations are involved in the normal formation of sperm pronuclei that subsequently ensure the successful fertilization.

Telomeric sequences play an important role in the reorganization and integration of sperm chromosomes (4, 5). They also conduct proper arrangement and separation of chromosomes during cell division, mitosis and meiosis (6, 7). Probably migration of telomeric chromatin to the cell membrane during spermatogenesis establishes unique architecture in the human sperm nucleus that are important in early chromatin remodeling at fertilization and early stages of fetal development (8-14).

To fulfill these roles, some features clearly distinguish telomeres of somatic cells with sperm. For example, unlike other mammals, 10-15% of the histones remain in human sperm (15-17). It is assumed that the remaining histones in human sperm tag specific genes for early expression in embryo (18); however, no evidence of nucleosomal ladder has been observed yet (19, 20).

In addition, numbers of testis-specific histone variants preferentially accumulate in telomeres (21-23). Even though this issue is not conserved, specific histone variants can organize particular regions of the genome, like telomeres, within the globally protamine- packaged sperm chromatin (24). Moreover, telomere-binding protein complex in human spermatozoa is different from somatic cells and contains telomeric histones like the H2B family, member W (*H2B.W*) (8, 25).

The H2B family, member W (*H2B.W*) gene, is one of the testis specific histone variant genes located at Xq22.2. *H2B.W* consists of three exons and two introns, expressed in particular stage of spermatogenesis (spermiogenesis). *H2B.W* is also present in mature sperm (21).

*H2B.W* causes chromatin fibers to resist against compaction (26). This special structure of chromatin may explain the dynamic rearrangement of telomeres at late stages of spermatogenesis, especially telomere extension occurring within elongating spermatids (27). This rearrangement can be a decisive factor to determine the position of telomeres in specific regions of mature sperm nucleus (28). These evidences suggest that *H2B.W* may also be a epigenetic marker necessary to identify and to cause the transmission of the telomeric chromatins through generations (29). Therefore, it is important to study the characterization of human telomere structure by *H2B.W* involvement to understand the mechanisms of fertilization (26).

*H2B.W* causes chromatin fibers to resist against compaction (26). This special structure of chromatin may explain the dynamic rearrangement of telomeres at late stages of spermatogenesis, especially telomere extension occurring within elongating spermatids (27). This rearrangement can be a decisive factor to determine the position of telomeres in specific regions of mature sperm nucleus (28). These evidences suggest that *H2B.W* may also be a epigenetic marker necessary to identify and to cause the transmission of the telomeric chromatins through generations (29). Therefore, it is important to study the characterization of human telomere structure by *H2B.W* involvement to understand the mechanisms of fertilization (26).

According to recent studies, copy number variations of *H2B.W* locus with other genes [plectin (*PLEC*), tetraspanin 7 (*TSPAN7*) and p21 protein (Cdc42/Rac)- activated kinase 3 (*PAK3*)] were found not only in men with sertoli cell only syndrome (SCOS), but also in women suffering from premature ovarian failure (POF) and XY gonadal dysgenesis. These 5 genes may imply a common genetic origin of lack of spermatogonia in the male and loss of oogonia in the female, leading to SCOS, XY gonadal dysgenesis and POF, respectively (30).

In addition, several studies indicated that genetic polymorphisms may also increase susceptibility to some forms of male infertility; for example, two recent allelic association studies on −9C>T (rs7885967) and 368A>G (rs553509) polymorphisms in *H2B.W* gene among different populations suggested that genetic variations of this gene could influence the susceptibility to spermatogenesis impairment (31, 32).

In this study, two single nucleotide polymorphisms (SNPs), -9C> T and 368A> G of *H2B.W* gene that may interfere in spermatogenesis were investigated in 152 Iranian men with known fertility status (92 infertile men with azoospermia and severe oligozoospermia and 60 fertile men with normal spermatogenesis) using polymerase chain reaction restriction fragment length polymorphism (PCR-RFLP) technique.

## Materials and Methods

### Participants

In this case controlled study conducted in Royan institute, ninety-two infertile men, aged from 25 to 46 years, presenting azoospermia and severe oligozoospermia were enrolled. Comprehensive characterizations of all patients including at least two semen analyses, physical examination, chromosome analysis and molecular tests were performed. Azoospermic group (n=46) were divided into three subgroups according to their testicular biopsy including patients with SCOS (n=21), complete maturation arrest (CMA, n=17) and hypo spermatogenesis (n=8). Severe oligozoospermic group (n=46) were defined with sperm count less than 5 million cells/mL.

Patients with history of cystic fibrosis, trauma, malignancies, varicocele, diabetes mellitus, hypertension, and chemotherapy were not included. Patients with Klinefelter syndrome, azoospermia factor (AZF) genes micro deletions or any identifiable cause of male infertility, including congenital bilateral absence of vas deference (CBAVD), were also excluded from the study groups by review of their records. Controls included healthy, fertile men, with at least one child within 3 years by spontaneous pregnancy and no history of miscarriage. The mean age of control group was 24 to 46 years. All donors gave an informed consent form before participation. The nationality of all groups was Iranian. All samples were collected during four-year period (2010-2013). This study was approved by the Ethical Committee of Royan Reproductive and Biomedicine Research Center.

### DNA preparation

The genomic DNA was extracted from the peripheral blood samples of each patient using salting- out method, according to the protocol (33).

### Choice of SNPs

**Fig.1:**
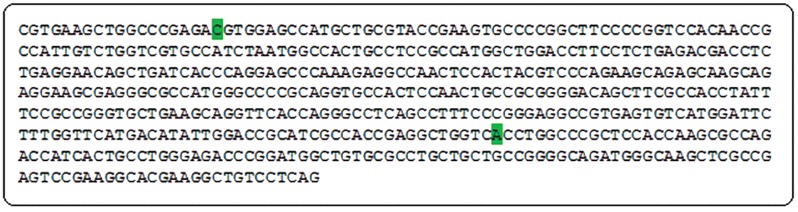
The sequence of 5′UTR and exon 1 of *H2B.W* gene. The position of the SNPs −9C>T and 368A>G are highlighted in green. SNP; Single nucleotide polymorphisms and UTR; Un-translated region.

Two SNPs in *H2B.W* gene that reportedly impact the impaired spermatogenesis were chosen for genotyping analysis including SNP −9C>T (rs553509) located in 5΄ un-translated region (5′UTR) and SNP 368A>G (rs7885967) with a missense mutation in exon 1 (32), which was in contrast to National Center for Biotechnology Information (NCBI) that refers to 368G>A, according to diverse allele distribution of SNPs in different populations. The sequence of normal *H2B.W* gene (NC_000023) was obtained from the NCBI website: http://www.ncbi. nlm.nih.gov (Fig.1).

### Polymerase chain reaction

Amplification of a fragment containing each of these SNPs was carried out by PCR according to the protocol of Ying et al. (32). PCR amplifications were performed in a final volume of 25 μl containing about 100 ng of extracted DNA, 200 μmol/L dNTPs, 10 pmol of each primer, 2.5 μl 10X PCR buffer, 1.5 mmol/l MgCl_2_ and 1.5 U Taq polymerase (CinnaGen, Tehran, Iran). PCR reaction consisted of an initial denaturing step at 95˚C for 5 minutes followed by denaturation at 95˚C for 45 seconds; annealing at 54˚C (−9C>T) and 60˚C (368A>G), respectively, for 45 seconds; extension at 72˚C for 35 seconds for 30 cycles; and a final extra extension at 72˚C for 10 minutes. Specific primer pairs used in these reactions are shown in table 1.

**Table 1:** Sequences of oligonucleotide primers used for genotyping analysis of *H2B.W*


	Forward primer	Reverse primer

5'UTR	5'-CATCCAATCAGACGTGAAGCTGGCCCGTGA-3'	5'-TGCTTCTGGGACGTAGTGGA-3'
Exon 1	5'-GTCTGGTCGTGCCATCTAAT-3'	5'-TACCTGAGGACAGCCTTCGT-3'


UTR; Un-translated region.

### Restriction enzyme treatment

For the next step, amplified fragments were digested overnight with position specific restriction enzymes. Restriction enzyme Tsp451 was used for genotyping analysis of −9C>T and Eco911 for 368A>G loci according to the manufacturer’s protocols (Fermentas, Vilnius, Lithuania).

### Electrophoretic separation

**Fig.2:**
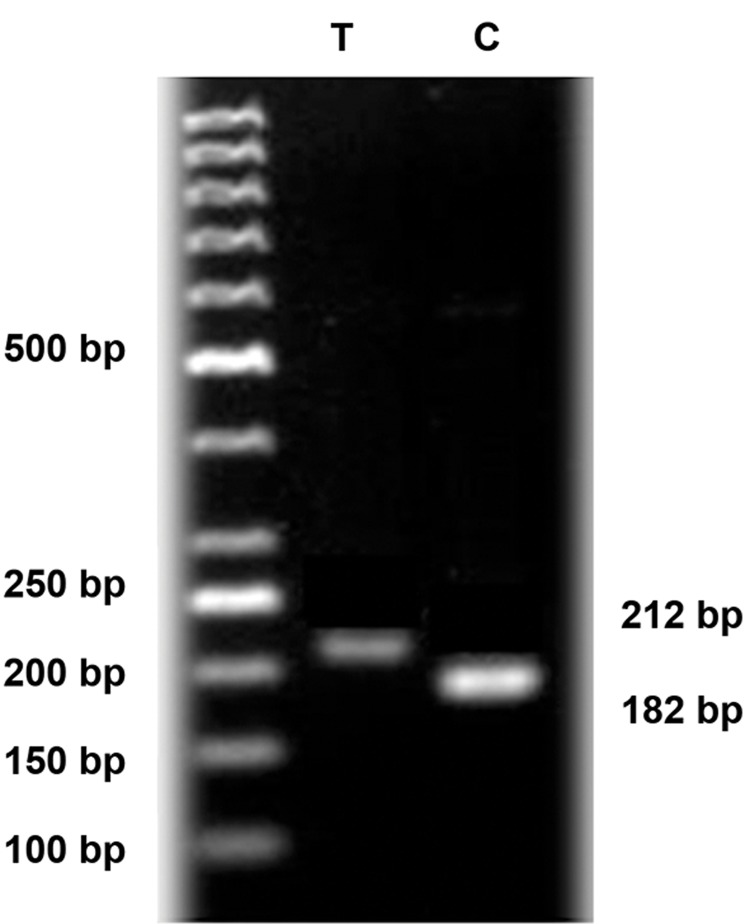
Restriction enzyme digestion of single nucleotide polymorphisms (SNP) −9C>T of polymerase chain reaction (PCR) product (30 bp band for −9C allele not shown in figure). The marker is a 50bp ladder.

**Fig.3:**
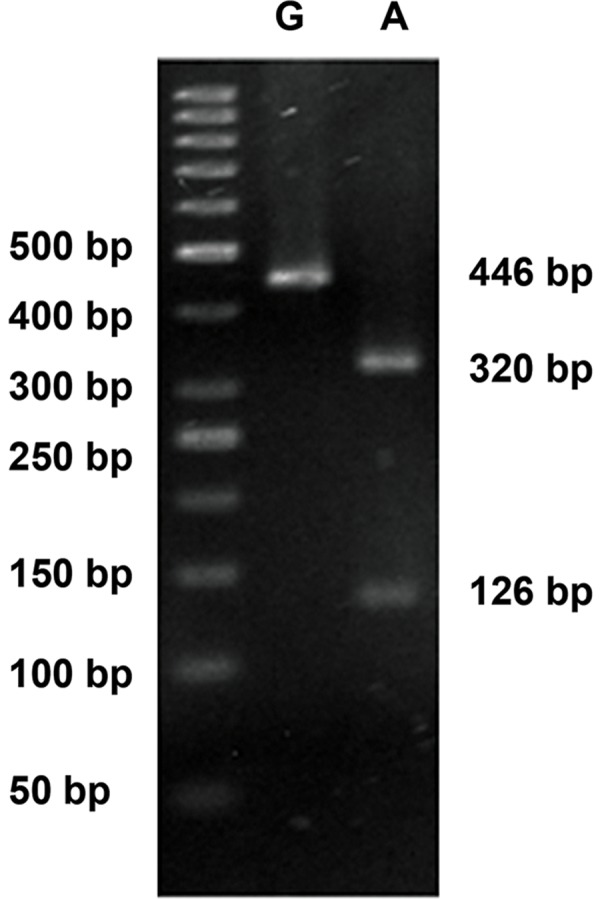
Restriction enzyme digestion of single nucleotide polymorphisms (SNP) 368A>G of PCR product. The marker is a 50bp ladder.

Electrophoretic separation were done by 3% agarose gel which indicated 212 bp band for allele T and two bands including 182 bp and 30 bp for allele C of −9C>T locus. Also visualization of 368A>G locus suggested two bands (320 bp and 126 bp) for Allele A and one band (446 bp) for allele G. The representative results of allele analysis for 368A>G and −9C>T loci in *H2B.W* gene by electrophoresis were shown in figures 2 and 3, respectively

**Fig.4:**
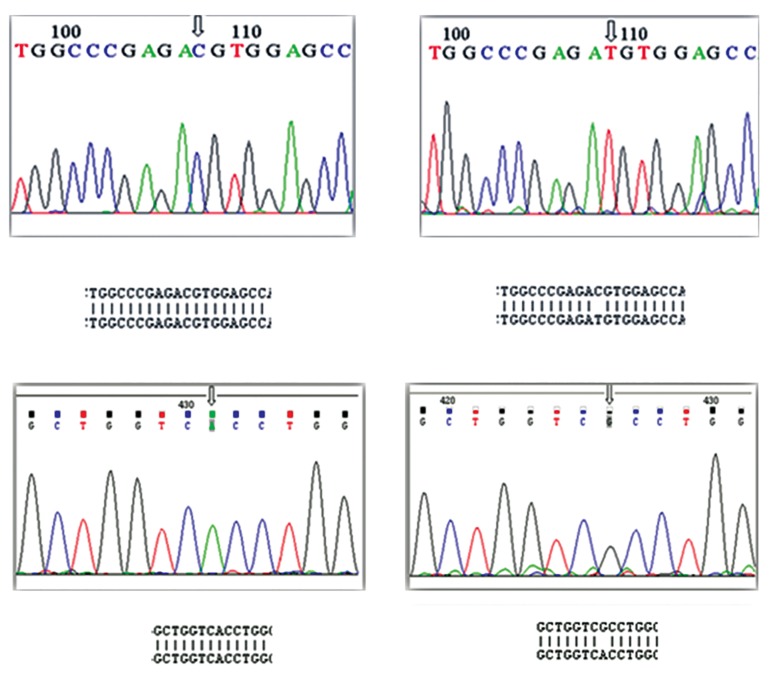
Direct sequencing of the polymerase chain reaction (PCR) products of 5′UTR and exon 1 of *H2B.W* gene. Arrow marks the sequences of −9C>T and 368A>G polymorphisms. UTR; Un-translated region.

Subsequently genotype alterations of some samples were confirmed performing direct DNA sequencing (Pishgam Biotech, Tehran, Iran). The reaction was carried out by Sanger method using ABI 3730xl capillary DNA sequencer (Fig.4).

### Statistical analysis

All the statistical analyses were performed using the Statistical Package for the Social Sciences (SPSS, SPSS Inc., Chicago, IL, USA). In this study, the genotype frequencies of control and patient groups were compared using the chi-square test. The P value lower than 0.05 was set as statistically significant. A logistic regression analysis was performed to calculate the odds ratio (OR) and 95% confidence interval (95% CI) for measuring the relation of alleles and haplotypes of two SNPs with male infertility.

### Results

This study investigated genetic alterations of two SNPs loci, −9C>T and 368A>G, in *H2B.W* gene in 92 infertile patients with azoospermia and severe oligozoospermia and 60 fertile men using a PCR-based RFLP analysis. The distribution frequencies of the two SNPs loci in azoospermic group or severe oligozoospermic subgroups and controls are listed in table 2. As shown in table 2, the frequencies of allele −9T of SNP −9C>T and allele 368G of SNP 368A>G in total patients, azoospermic and severe oligozoospermic patients, had no significant difference in contrast with controls (P>0.05).

In subgroups of azoospermia patients, the frequency of allele −9T in patients suffering from CMA was significantly higher compared with patients suffering from SCOS (P=0.015). The distribution frequencies of the two SNPs loci in azoospermic subgroups are listed in table 3. Also the allele frequency distributions between azoospermic subgroups and controls are shown in table 4.

Four kinds of haplotypes of the two SNPs (CA, TA, CG, and TG) were observed in both infertile patients and controls. Accordingly the haplotype TA compared with haplotype CA significantly increased in patients suffering from CMA, compared with men had SCOS (P=0.029). Tables 5-7 show the results of haplotypes observations.

**Table 2:** The distributions of allele frequencies of SNPs −9C>T and 368A>G in *H2B.W* gene in infertile patients with azoospermia or sever oligozoospermia and fertile men


Locus		Fertile men	Infertile patients			P value^a^			OR (95% CI)^a^		
	Allele	Total (n=60)	Total (n=92)	Azoospermia(n=46)	Severe oligo zoospermia(n=46)	1	2	3	1	2	3

-9C>T	C	58.30%(n=35)	59.80%(n=55)	65.2%(n=30)	54.3% (n=25)	0.859	0.471	0.682	0.942(0.486-1.824)	0.747 (0.337-1.653)	1.17(0.542-2.550)
	T	41.70%(n=25)	40.20%(n=37)	34.7%(n=16)	45.7% (n=21)						
368A>G	A	63.30%(n=38)	65.20%(n=60)	65.2%(n=30)	65.2% (n=30)	0.812	0.841	0.841			
	G	36.70%(n=22)	34.80%(n=32)	34.7%(n=16)	34.8% (n=16)				0.921(0.468-1.815)	0.921 (0.413-2.055)	0.921(0.413-2.055)


SNP; Single nucleotide polymorphisms, OR; Odd ratio, CI; Confidence interval, ^a^; Controls vs. 1; Total infertile patients, 2; Azoospermia, and 3; Severe oligozoospermia. Due to the fact that *H2B.W* is on the X chromosome and that the subjects studied are 46, XY, there are no heterozygous men with both alleles (-9C and -9T; 368G and 368A).

**Table 3:** The distributions of allele frequencies of SNP −9C>T and 368A>G in *H2B.W* gene in azoospermia according to testicular biopsy


Locus		Azoospermia (n=46)			P value^a^			OR (95% CI)^a^		
	Allele	Men with hypospermatogenesis(n=8)	CMA(n=17)	SCOS (n=21)	1	2	3	1	2	3

-9C>T	C	75%(n=6)	41.20%(n=7)	81%(n=17)	0.127	0.724	0.015	4.286(0.661-27.78)	0.706(0.102-4.891)	0.165(0.038-0.706)
	T	25%(n=2)	58.80%(n=10)	19.00%(n=4)						
368A>G	A	87.50%(n=7)	70.60%(n=12)	52.40%(n=11)	0.37	0.109	0.257	2.917(0.281-30.290)	6.634(0.662-61.199)	2.182(0.566-8.414)
	G	12.50%(n=1)	29.40%(n=5)	47.60%(n=10)						


SNP; Single nucleotide polymorphisms, CMA; Complete maturation arrest, SCOS; Sertoli cell only syndrome, OR; Odd ratio, CI; Confidence interval, ^a^; Men with hypo spermatogenesis vs. 1; CMA, 2; SCOS and 3 SCOS vs. CMA.

**Table 4:** The distributions of allele frequencies of SNPs −9C>T and 368A>G in *H2B.W* gene in azoospermia according to testicular biopsy and fertile men


Locus		Fertile men	Azoospermia (n=46)			P value^a^			OR (95% CI)^a^		
	Allele	Total(n=60)	Men with hypospermatogenesis(n=8)	CMA(n=17)	SCOS (n=21)	1	2	3	1	2	3

-9C>T	C	58.30%(n=35)	75%(n=6)	41.20%(n=7)	81%(n=17)	0.374	0.214	0.071	0.467(0.087-2.505)	2(0.670-5.971)	0.329(0.099-1.098)
	T	41.70%(n=25)	25%(n=2)	58.80%(n=10)	19.00%(n=4)						
368A>G	A	63.30%(n=38)	87.50%(n=7)	70.60%(n=12)	52.40%(n=11)	0.144	0.364	0.636	0.2(0.023-1.729)	0.583(0.182-1.866)	1.273(0.469-3.454)
	G	36.70%(n=22)	12.50%(n=1)	29.40%(n=5)	47.60%(n=10)						


SNP; Single nucleotide polymorphisms, CMA; Complete maturation arrest, SCOS; Sertoli cell only syndrome, OR; Odd ratio, CI; Confidence interval, ^a^; Controls vs. 1; Men with hypo spermatogenesis, 2; CMA and 3; SCOS.

**Table 5:** The distributions of haplotype frequencies of SNPs −9C>T and 368A>G in *H2B.W* gene in infertile patients with azoospermia or sever oligozoospermia and fertile men


Haplotype	Fertile men	Infertile patients			P value^a^			OR (95% CI)^a^		
	Total (n=60)	Total (n=92)	Azoospermia(n=46)	Severe oligozoo spermia (n=46)	1	2	3	1	2	3

CA	33.30%(n=20)	30.40%(n=28)	34.70%(n=16)	26.00%(n=12)	0.693	0.756	0.688	Reference	Reference	Reference
TA	30%(n=18)	34.80%(n=32)	30.40%(n=14)	39.10%(n=18)	0.565	0.954	0.302	1.27(0.563-2.866)	0.972(0.373-2.573)	1.667(0.632-4.392)
CG	26.70%(n=16)	29.30%(n=27)	30.40%(n=14)	28.20%(n=13)	0.664	0.857	0.562	1.205(0.519-2.802)	1.094(0.413-2.894)	1.354(0.487-3.769)
TG	10%(n=6)	5.40%(n=5)	4.30%(n=2)	6.50%(n=3)	0.441	0.321	0.819	0.595(0.159-2.224)	0.417(0.074-2.350)	0.883(0.175-3.965)


SNP; Single nucleotide polymorphisms, OR; Odd ratio, CI; Confidence interval, ^a^; Controls vs. 1; Total infertile patients, 2; Azoospermia and 3; Sever oligozoospermia.

**Table 6:** The distributions of haplotype frequencies of SNPs −9C>T and 368A>G in *H2B.W* gene in azoospermia according to testicular biopsy


Haplotype	Azoospermia (n=46)			P value^a^			OR (95% CI)^a^		
	Men with hypospermatogenesis(n=8)	CMA(n=17)	SCOS (n=21)	1	2	3	1	2	3

CA	62.50%(n=5)	17.60%(n=3)	38.10%(n=8)	0.246	0.519	0.104	Reference	Reference	Reference
TA	25%(n=2)	52.90%(n=9)	14.30%(n=3)	0.06	0.952	0.029	7.5(0.921-61.047)	0.937(0.114-7.728)	0.125(0.019-0.805)
CG	12.50%(n=1)	23.50%(n=4)	42.90%(n=9)	0.155	0.15	0.851	6.6670.487-91.331)	5.625(0.537-58.909)	0.844(0.143-4.974)
TG	0	5.90%(n=1)	4.80%(n=1)	1	1	0.532	2.69E+09(0.000- .)	1.01E+09(0.000-.)	0.375(0.017-8.103)


SNP; Single nucleotide polymorphisms, CMA; Complete maturation arrest, SCOS; Sertoli cell only syndrome, OR; Odd ratio, CI; Confidence interval, ^a^; Men with hypo spermatogenesis vs. 1; CMA, 2; SCOS and 3; SCOS vs. CMA.

**Table 7:** The distributions of haplotype frequencies of SNPs −9C>T and 368A>G in *H2B.W* gene in azoospermia according to testicular biopsy and fertile men


Haplotype	Fertile men	Azoospermia (n=46)			P value^a^			OR (95% CI)^a^		
	Total(n=60)	Men with hypospermatogenesis(n=8)	CMA(n=17)	SCOS (n=21)	1	2	3	1	2	3

CA	33.30%(n=20)	62.50%(n=5)	17.60%(n=3)	38.10%(n=8)	0.602	0.359	0.358	Reference	Reference	Reference
TA	30%(n=18)	25%(n=2)	52.90%(n=9)	14.30%(n=3)	0.366	0.104	0.244	0.444(0.077-2.581)	3.333(0.779-14.26)	0.417(0.096-1.815)
CG	26.70%(n=16)	12.50%(n=1)	23.50%(n=4)	42.90%(n=9)	0.226	0.54	0.564	0.25(0.026-2.361)	1.667(0.325-8.549)	1.406(0.442-4.473)
TG	10%(n=6)	0	5.90%(n=1)	4.80%(n=1)	0.999	0.933	0.45	0(0.000--)	1.111(0.097-12.750)	0.417(0.043-4.034)


SNP; Single nucleotide polymorphisms, CMA; Complete maturation arrest, SCOS; Sertoli cell only syndrome, OR; Odd ratio, CI; Confidence interval, ^a^; Controls vs. 1; Men with hypo spermatogenesis, 2; CMA and 3; SCOS vs. CMA.

## Discussion

Study mutations in human X-linked genes with a testis-specific pattern in view of male infertility are considered to be remarkable. Firstly this chromosome is enriched for genes expressed in reproduction-related tissues and secondly it is due to its hemizygous exposure in men (34). *H2B.W* is a newfound X-linked gene that its characteristic and association with male infertility have been reported recently.

In this study, the prevalence of two SNPs −9C>T and 368A>G, in *H2B.W* genes, was conducted on a population of Iranian infertile men.

The present study showed that the frequency of −9T at the −9C>T locus was significantly higher in CMA group than in patients with SCOS (Table 3), suggesting that the mutation of allele C to T in *H2B.W* gene might influence mRNA stability or its overall translation rate (35) that leads to arrest the maturation process of spermatids. This could also explain the expression of *H2B.W* gene at late stages of spermatogenesis.

As shown in table 2, in general, no significant differences are found in the frequencies of −9T allele between two groups of controls and patients, proposing that the alteration of allele C to T may be insufficient reasons for infertility in Iranian men. In contrast to previous studies, −9C>T polymorphism is associated with spermatogenic impairment in South Korean and Chinese populations (31, 32) which may be related to the following factors: environmental factors, characteristics of subjects, as well as X chromosome haplogroups in different ethnic populations.

In addition no notable association between SNP 368A>G and the risk of male infertility in Iranian population was found in this investigation. These results are similar to study on South Korean population (31).

Finally, haplotype analysis of patient and control groups was performed in *H2B.W* gene. The results of haplotype analysis showed that the haplotype TA compared with haplotype CA significantly increased in patients suffering from CMA, compared with men had SCOS, suggesting that haplotype TA might arrest maturation process of spermatids during spermatogenesis. In fact patients with TA haplotype seem to be at higher risk of azoospermia caused by CMA of spermatids. Haplotype analysis in the study by Ying et al. also suggested that haplotype CA may be a protection factor from spermatogenesis disorder and haplotype TG may be a risk factor for azoospemia or oligozoospermia (32).

Therefore, it may be suggested that SNPs −9C>T and 368A>G of *H2B.W* genes have no crucial roles in spermatogenic failure in the Iranian population, except those observed in patients suffering from CMA.

To further study, it would be better to investigate the expression level of *H2B.W* in testis tissue of patients with −9T polymorphism, but unfortunately in our study, their tissues were not available.

## Conclusion

The present study showed no significant correlation of SNPs −9C>T and 368A>G in *H2B.W* gene with susceptibility to spermatogenesis impairment in Iranian men, although it could be presumed that allele −9T in 5'UTR of *H2B.W* gene arises the risk of complete maturation arrest in azoospermic patients. Also this study indicated that haplotype CA compared to haplotype TA might be a protective factor for maturation process of spermatids. Further studies in larger size samples are needed to assessment the exact role of *H2B.W* gene in sperm nucleus.
